# Unveiling neurocysticercosis: A call for heightened awareness and action

**DOI:** 10.1016/j.bas.2024.104174

**Published:** 2024-12-24

**Authors:** Inibehe Ime Okon, Muhammad Danish Shafqat, Muhammad Daniyal Shafqat, Javeria Hussain, Youssef Razouqi

**Affiliations:** a Benjamin S. Carson (Snr) College of Health and Medical Sciences, Babcock University, IIishan-Remo, Ogun State, 121003, Nigeria; b Department of Neurosurgery, Dell Medical School, University of Texas at Austin, TX, 78712, United States; c Department of Surgery, Shifa College of Medicine, Islamabad, Pakistan; d Interdisciplinary Laboratory of Biotechnology and Health, Neurosciences and Cellular Physiology research team, Mohammed VI University of Sciences and Health (UM6SS), Casablanca, Morocco

Dear Editor,

The authors write to bring attention to a critical yet often overlooked aspect of global health, and surgery. Initially, we would like to commend the substantive content of the article, particularly its documentation of a successful and timely neurosurgical intervention in a challenging case. Furthermore, we applaud the attention drawn to the issue of neurocysticercosis (NCC), a neglected parasitic disease, with an estimated prevalence of 22% in people in epilepsy in sub-Sahara Africa according to a 2020 meta-analysis (Owolabi et al., 2020). NCC is a pleomorphic disease linked to a range of manifestations. As such, recognizing the complexity of diagnosing this uncommon disease is essential. In particular, we acknowledge the importance of maintaining NCC as a potential consideration in the differential diagnosis of cranial lesions in Africa. While we commend the authors for their efforts in addressing the concerns related to this disease, we believe it pertinent to discuss several critical aspects of this disease, including its clinical manifestations, diagnostic modalities, and propensity for recurrence, and intend to emphasize these points in the hope that they may facilitate early detection by healthcare practitioners, particularly in low socio-economic regions with a high prevalence.

Neurocysticercosis, a parasitic infection of the nervous system, manifests with a diverse array of symptoms, which depend on the location and number of cysts within the brain. Seizures are the most prevalent symptom, affecting 70%–90% of symptomatic individuals, according to recent research (Steyn et al., 2022). These seizures can be partial or generalized and may occur once or recur over time. While some patients remain asymptomatic, adding complexity to the diagnosis, the presence of seizures and headaches should prompt a high index of suspicion (Saito et al., 2017). Recurrent and severe headaches also serve as a key symptom. A recent case report by Byrnes and colleagues described neurocysticercosis in a middle-aged man with a long history of migraines (Byrnes et al., 2024). The authors emphasized how these nonspecific symptoms can be overlooked by physicians, underlining the importance of considering this condition when an established neuropathological condition changes in presentation or when modifications in ongoing therapy are necessary, regardless of obvious risk factors.

The development of intracranial hypertension is a severe complication of NCC, caused by the obstruction of cerebrospinal fluid flow. This condition may provoke symptoms such as headaches, nausea, and vomiting. In severe cases, emergency intervention to reduce intracranial pressure is required, often through the administration of steroids, especially given the high mortality rates associated with extraparenchymal neurocysticercosis (Fogang et al., 2015).

Moreover, the number, size and location of cysts within the brain can lead to a variety of focal neurological deficits, such as gait abnormalities, visual disturbances and alterations in mental state including drowsiness and psychomotor retardation (Fogang et al., 2015; Mustafa et al., 2020; Shafqat and Okon, 2024; James et al., 2024; Ojo et al., 2024). Bruns syndrome, a rare presentation first identified in 1902, features a sudden onset of severe headache, nausea and vomiting, along with a vestibular syndrome triggered by rapid head movements, due to cysts in the ventricular system. These cysts can also lead to hydrocephalus (Roque et al., 2021), requiring timely neurosurgical intervention. Despite the wide spectrum of symptoms, it is critical to acknowledge that many patients may be asymptomatic, which further complicates the diagnostic process.

In diagnostic approaches in low socio-economic areas, neurocysticercosis is particularly prevalent in the developing world, as recognized by the World Health Organization, yet it remains a neglected disease due to limited awareness and understanding. In low socioeconomic regions, diagnosing neurocysticercosis (NCC) presents a significant challenge due to restricted access to advanced imaging technologies. However, resourceful and cost-effective diagnostic methods can still be effectively utilized. Skilled healthcare providers perform a detailed medical history and a comprehensive neurological examination, which form the cornerstone of the initial diagnostic approach. Studies have shown that experienced clinicians can achieve high diagnostic accuracy for NCC through these evaluations, especially when supplemented by neuroimaging in endemic areas (White et al., 2018). Enzyme-linked immunosorbent assay (ELISA) tests are promising for detecting antibodies specific to the parasite that causes neurocysticercosis. However, the Enzyme-Linked Immunoelectrotransfer Blot (EITB) assays are recommended due to their superior sensitivity and specificity. Nonetheless, both assays can serve as effective screening tools in high-risk areas (Proaño et al., 2002; Garvey et al., 2017).

Furthermore, neuroimaging is an indispensable tool for obtaining a definitive diagnosis of neurocysticercosis. While magnetic resonance imaging (MRI) is considered the gold standard, its availability may be limited in low-resource settings. In such scenarios, computed tomography (CT) scans provide a valuable alternative (Garcia et al., 2014). A combined diagnostic strategy that includes clinical evaluation, serological testing such as ELISA, and CT imaging when available, offers a pragmatic approach for diagnosing NCC in resource-limited environments. Nevertheless, diagnosis has to be prompt and accurate to facilitate timely intervention and prevent potential complications.

Finally, delay in diagnosing and poor management of NCCs could lead to recurrence of the disease. The likelihood of recurrent neurocysticercosis is influenced by multifactorial determinants, with some studies indicating its occurrence in a significant proportion of patients. An investigation conducted in India, documented in a 2021 publication, revealed a recurrence rate of 12.6% (15 out of 119 patients) over a follow-up period ranging from 14 to 25 months (Kaur et al., 2021). Similarly, a retrospective cohort study conducted over a decade at a prominent academic center in the USA reported a recurrence rate of 17% (Ramanathan et al., 2021). Notably, the presence of degenerating or residual calcified cysticercus cysts within the brain parenchyma is identified as the primary risk factor for NCC recurrence, as elucidated by Singh and colleagues (Singh et al., 2013). Additional contributors to recurrence include incomplete treatment, compromised immune response, and reinfection. Vigilant neuroimaging surveillance, as demonstrated in the referenced case report, and a heightened index of suspicion are imperative for the expeditious management of NCC.

Although there is a plethora of literature detailing recommended treatment modalities, Hamamoto Filho and colleagues described various cyst presentations within the brain and corresponding treatment approaches, including medical management, follow-up protocols, and surgical interventions such as for ventricular cysts (Hamamoto et al., 2022). Such comprehensive insights underscore the necessity for tailored therapeutic strategies aligned with the distinct clinical manifestations of neurocysticercosis.

In closing, neurocysticercosis remains a significant public health challenge, particularly in endemic regions where recurrence rates are notable. It is essential that treatment and surveillance protocols be dynamically tailored to address the multifactorial determinants of recurrence, such as incomplete treatment and compromised immune response. Furthermore, the development and implementation of rigorous neuroimaging and surveillance strategies are vital for early detection and management of recurrent cases. The insights from recent studies and literature on treatment modalities serve as a foundation for advancing targeted therapeutic strategies. These strategies should be specifically designed to accommodate the varied clinical manifestations of neurocysticercosis, ultimately improving patient outcomes and reducing the burden of this disease on affected populations.

## Funding statement

None.

## Declaration of Competing interests

Not applicable.
